# Blame or Justification: A Conceptual Model of How Attributions Shape Observers’ Deviant Responses to Abusive Supervision

**DOI:** 10.3390/bs16071191

**Published:** 2026-07-15

**Authors:** Merve Ağaoğlu, Oya Erdil

**Affiliations:** 1 Faculty of Business Administration, Gebze Technical University, Kocaeli 41400, Türkiye; erdil@gtu.edu.tr; 2 Graduate School, Istanbul Technical University, Istanbul 34467, Türkiye

**Keywords:** attribution theory, power distance, third-party observers, vicarious abusive supervision, workplace deviance

## Abstract

The literature on antisocial bystander responses to vicarious mistreatment predominantly considers observer deviance as a retaliatory, emotion-based behavior and examines it through the lens of deontic justice. This perspective centers on the idea that observers are disturbed by abuse and act in favor of the victim with a desire to restore justice. However, recent research indicates that observers may also engage in deviance towards the victim, which makes the deonance theory insufficient for explaining different forms of bystander aggression. Therefore, to provide further insights into bystander aggression directed at the victim, we need to rely on an alternative perspective. Grounded in attribution theory, this work approaches observer deviance not as an emotional response but as a result of a cognitive process and proposes an attribution-driven model. We discuss the observer attribution process as an antecedent to different types of observer deviance that emerge following vicarious abusive supervision. Drawing on research on observers’ deviant reactions following vicarious abusive supervision, we conceptualize observers’ behaviors into two main categories (i.e., reciprocity-based and victim-based) and identify two main attribution types (i.e., responsibility and justification) that observers can make. Consequently, we develop a conceptual model proposing the mediating effects of observer attributions. Additionally, we discuss the possible moderating effects of power distance— at both the individual and the organizational levels—that may influence observer evaluations of coworker abuse. Our model addresses the role of observers’ evaluations of vicarious mistreatment, offers several empirically testable research propositions, and enhances our understanding of third-party deviance.

## 1. Introduction

Destructive employee behaviors (e.g., workplace antisocial behaviors, counterproductive work behaviors, deviant workplace behaviors) are important phenomena that can cause a negative impact on organizational sustainability and well-being; therefore, there is an effort by researchers to understand their dynamics. Workplace deviance, which we will focus on, is characterized by voluntarily performed harmful behaviors that violate organizational norms (Robinson & Bennett, 1995) and represents a consequence of abusive supervision. Research in the field indicates that both victims (e.g., Lian et al., 2014; C. Liu et al., 2021) and observers (e.g., Mitchell et al., 2015; Chen & Liu, 2019) of abusive supervision may engage in deviant behaviors towards the perpetrator as a response. Additionally, different agents (e.g., the organization itself, customers, coworkers) may also be targets of this aggression (see C. Liu et al., 2021; H. Wang & Xiao, 2021; Dhanani & LaPalme, 2019). In other words, abusive supervision may lead to a hostile organizational climate through negative behaviors enacted by both the victim and the observer, and directed toward different targets, not only the perpetrator. In this context, there is an increase in research on third-party harmful responses to vicarious abusive supervision (e.g., B. Ma et al., 2017; Wei et al., 2023), particularly in recent years.

In the relevant literature, bystander reactions to workplace mistreatment have predominantly been examined through the lens of deonance theory (for details, Folger, 2001; Cropanzano et al., 2003), which sees people’s reactions as deontic reactions that are motivated through emergent emotions after witnessing a moral violation. It is also a common view that bystander deviance, motivated by these emotions, is retaliatory (e.g., Chen & Liu, 2019). However, some studies’ results indicate that observers of mistreatment can also develop destructive behaviors directed at the victim (e.g., Xu et al., 2020; Bai et al., 2022; P. Liu et al., 2022; Hill et al., 2025), which makes the deontic justice perspective insufficient as a theoretical explanation of observer aggression. Although there are other theories that attempt to explain observer mistreatment towards peers (e.g., social learning, social identity, sensemaking), these theories cannot provide an integrated framework for explaining the development of different types of deviance.

As Skarlicki and Rupp (2010) underline, decisions and perspectives on justice judgments are also influenced by cognitive processes (e.g., social reasoning) as well as emotional processes. Whether observer aggression is motivated by a desire for justice or by an effort to adapt to the social context, it is appropriate to analyze this phenomenon under a cognitive evaluation process. Furthermore, according to Skarlicki (2001), culture can influence employee interpretations of actions or justice perceptions. A workplace incident that would provoke anger in a particular cultural context might be considered reasonable in another context; therefore, it may not trigger any feelings. Consequently, a theoretical foundation that focuses on cognition, rather than emotion, may provide a more adaptable perspective for third-party evaluations of workplace mistreatment in different cultural settings. For example, Shoss et al. (2013) state that attribution processes in workplaces may show similarities across different cultural contexts. In line with these arguments, we argue that third-party deviance is a consequence of a cognitive process (i.e., attribution process), distinguishing it from an emotionally driven deontic response.

The attribution process refers to the cognitive reasoning assessment that the social subject makes to answer the “why” question (Kelley, 1973). In other words, attributions are individuals’ perceived explanations for events. Numerous studies have benefited from attribution theory for understanding workplace incidents. One example is Bowling and Beehr’s (2006) workplace attribution model, which posits that employees’ attributions (i.e., whom they find responsible for mistreatment) have an impact on their behavioral responses. Again, empirical findings show that (e.g., Bowling & Michel, 2011; Shoss et al., 2013) employees’ attributions may be associated with different types of negative behavior. Following a similar approach, we argue that observer attributions are a predictor of third-party deviance.

Drawing on the prior literature, we propose a conceptual model of the attribution process for vicarious abusive supervision. First, we introduce two directions (i.e., responsibility attribution versus justification attribution) as possible explanations for observers’ evaluations of abuse and discuss the implications of these attributions for different types of observer deviance. Since attributions are influenced by personal beliefs (Kelley & Michela, 1980), an attribution-based perspective that does not examine beliefs may lead to inadequate addressing of the subject. Moreover, employees’ outcomes may be influenced by individual and contextual factors. Thus, to increase the explanatory power of the model, we also argue the potential effects of power distance beliefs—acceptance of power inequalities (Hofstede, 1983; Zhang et al., 2010)—at both the individual and the organizational levels. Specifically, we discuss the potential effects of personal power distance values as a dispositional factor on observer attributions. In addition, we also add organizational power distance to the model as a contextual factor to see the possible effects on the different types of deviant behaviors. As perceptions of abuse can change according to employee acceptance of power inequalities (Lian et al., 2012; W. Wang et al., 2012; Zhang & Liao, 2015) and because organizational processes naturally lie in hierarchy and power differences (Lunenburg, 2012) between superior and subordinate, power distance is a meaningful variable in this work’s context.

Our paper extends prior research by adapting workplace attribution theory beyond victims to third parties and by examining bystander deviance as a cognition-focused behavior instead of an emotion-focused response. In this way, we suggest an explanation for bystander deviant behavior directed towards the victim—that retaliation does not provide an answer—and we propose an integrated model of observer deviance, previously explained through different theoretical perspectives. Additionally, by discussing the potential effects of individual and organizational values about the distribution of power on shaping employee perceptions and behaviors, we respond to numerous calls for additional research (e.g., Chen & Liu, 2019; Zhang & Liao, 2015) on the impact of cultural values on employee reactions to observed abusive supervision. Lastly, we also answer calls (Bowling & Beehr, 2006; Dhanani & LaPalme, 2019) for clearer explanations of observer attribution processes and calls for peer mistreatment (Bai et al., 2022) of the abused employee.

## 2. Literature Review

### 2.1. Observed Abusive Supervision and Third-Party Deviant Behavior

Workplace deviance is a type of antisocial work behavior (e.g., teasing, gossiping, ostracizing) that violates organizational norms (Bennett & Robinson, 2000). External factors, such as management styles or organizational climate, can lead to these undesirable workplace behaviors (Di Stefano et al., 2019). Studies (e.g., Lian et al., 2014; W. Wang et al., 2012) indicate that victims of toxic management styles, such as abusive supervision—verbal or nonverbal antagonistic actions in workplaces (non-physically; Tepper, 2000)—can engage in deviant behaviors. Moreover, these destructive consequences can extend to a wider area (Priesemuth, 2013) as employee behaviors may be influenced by their perceptions of how other group members are treated (Yang et al., 2007). In this context, there is growing research on observer attitudes in the abusive supervision process. While early studies focus on prosocial behaviors towards the victim (e.g., supporting, helping), recent studies show that observers can also respond to vicarious abusive supervision in an antisocial way. For example, relevant studies show that (Mitchell et al., 2015; Zhang et al., 2020) observers could engage in aggressive reactions (i.e., supervisor-directed deviance) toward the perpetrator if they felt anger about the abuse. Moreover, Dhanani and LaPalme (2019) underline that observers can engage in antisocial behaviors not only against the perpetrator but also against the organization itself.

Existing literature (e.g., O’reilly & Aquino, 2011; B. Ma et al., 2017) has mostly seen bystander deviances as retributive behaviors motivated by the re-organization of justice and has predominantly been analyzed from the deontic justice perspective (i.e., the idea that people are also concerned with others’ conditions, interests, and well-being). When people observe unfairness, they show deontic reactions through emerged emotions (e.g., sadness) to restore justice (Folger, 2001; Cropanzano et al., 2003). In other words, the main underlying motivation behind such observer deviance is supporting the victim. However, some studies (e.g., Xu et al., 2020; Bai et al., 2022; Hill et al., 2025) interestingly show that victims may also be exposed to harm and mistreatment by observers after being abused, which brings the idea that observers may not be acting in accordance with the norm of reciprocity, or may not always aim to protect the victim. That is, while the deontic justice approach explains observers’ aggression toward agents who are perceived as distorting justice, it fails to explain the antisocial behaviors directed at the victim.

Nevertheless, negative observer behaviors towards the victim are again predominantly analyzed through emotions. For example, Xu et al. (2020) explained the observer’s deviant behavior toward the victim as a result of triggered schadenfreude (i.e., the feeling of pleasure derived from others’ misfortune; van Dijk & Ouwerkerk, 2014) emotion. However, there are also a few studies examining the underlying mechanisms beyond emotions in observer mistreatment. For example, Bai et al. (2022) explained observer harassment towards the victim under the heading of the social learning mechanism (i.e., acquiring new behaviors through observation and imitation; Bandura, 1977). They argue that observers who engage in peer harassment against the victim may take their abusive leaders as role models as a result of their formal authority and learn from them to act this way. Another example is P. Liu et al.’s (2022) work, which explains the observer mistreatment toward the victim of workplace incivility using social identity theory. According to this study, as a result of observed leader incivility, the victim may be defined by the observer as “other” (i.e., perceived as an outsider), and this categorization may lead to deviance toward the victim.

Similar to these efforts, we discuss observer deviance within the context of a cognitive mechanism, not as a deontic emotional response. Based on previous works (see Bowling & Beehr, 2006; Harvey et al., 2014; Chen & Liu, 2019), we use attribution theory to propose a model that explains the emergence of different observer deviant behaviors. By doing so, we intend to offer an explanation for the occurring process of aggression that is not motivated by retaliation, and thus, is not fully captured by deontic theory.

### 2.2. Attribution Theory and Workplace Mistreatment

Deontic moral reasoning process takes place unconsciously and rapidly (Dhanani & LaPalme, 2019), but the attribution process is a more time-consuming reasoning process (Kelley & Michela, 1980). The attributions are our perceived explanations for actions that help us adapt to the environment (Weiner, 1985) and are associated with crucial workplace outcomes (Harvey et al., 2014) in organizations.

Attribution theory constitutes one of the applied theoretical perspectives in organizational behavior, particularly in the context of workplace mistreatment. One reason for using this theory is the effort to explain the victim’s harmful behaviors directed at different targets (e.g., the organization, a coworker). Generally, in accordance with the principle of reciprocity (i.e., the idea of “the return of injuries”; Gouldner, 1960), it is expected that the subject of the action develops a response to the other subject. That is, in the context of abusive supervision, the victim is expected to direct harmful behavioral responses (e.g., supervisor-directed deviance) toward the supervisor as payback. However, some studies indicate that (e.g., Sulea et al., 2013; C. Liu et al., 2021) different agents, such as the organization, may also be the target of the victim’s aggression. Among the efforts (e.g., displacing aggression; Mitchell & Ambrose, 2007) to explain this situation, attribution theory has also been used. For example, Bowling and Beehr (2006) proposed an attribution-based model of workplace harassment, focusing on victims’ causal attributions for workplace harassment. This model suggests that victims’ causal attributions—perceived explanations for actions related to workplace harassment—are antecedents of different responses. They argue that the outcomes directed at the organization may be reciprocity reactions, as the organization is held responsible for the abuse.

Despite not using attribution theory, similarly, Tepper et al. (2008) claim that harmful behaviors directed towards the organization among abused employees might be their beliefs about the organization’s responsibility for abusive supervision. Empirical findings provide evidence that victims may attribute responsibility for mistreatment not only to the perpetrator but also to others. For example, Breaux et al. (2010) examined employee attributions in abusive supervision from a justice perspective and found that victims might hold both organization and supervisor blameworthy. Bowling and Michel (2011) found that harmful actions (i.e., counterproductive work behavior) toward the organization were related more strongly among the employees who believed the organization was responsible for abuse than among employees who did not attribute the abuse to the organization.

In light of this research, we propose an observer attribution model below that explains different types of observer deviance both as a result of blame and justification attributions. In doing so, we hope to explain observer deviant behavior, which deontic justice cannot explain, and to create an integrated model for observers’ aggression examined from different perspectives.

## 3. Proposed Research Model and Proposition Development

Causal explanations are not only made by the parties involved in the action. Like the victims, bystanders’ judgments about an action may play a crucial role in their responses (Shao et al., 2026). As observers perceive abuse as a potential threat to their own well-being (Chen & Liu, 2019), they are also likely to evaluate the situation to identify the main cause of the abuse. Weiner (1979) states that the social subject seeks explanations to reach a prescription (Weiner, 1985) for their future actions. Because of the subjectivity inherent in attribution’s nature (Weiner, 1979), different people may perceive different reasons for a particular action. This explains the variability of both the attributions and the human behaviors influenced by them. That is, a person approaches most of the problems with their beliefs about related causes and effects (Kelley & Michela, 1980), which explains why observers may respond differently in the same situation.

Drawing on the relevant research of observer outcomes after vicarious abusive supervision, we examine observer deviance under two main categories: reciprocity-based and victim-based. As antecedents to these two behaviors, we propose two primary attributional directions (i.e., responsibility and justification) that observers can make, as follows. Before introducing these two concepts, it should be noted that responsibility attribution and justification attribution, discussed in detail below, represent two independent dimensions of the observer employee’s evaluative judgments.

**Figure 1:**
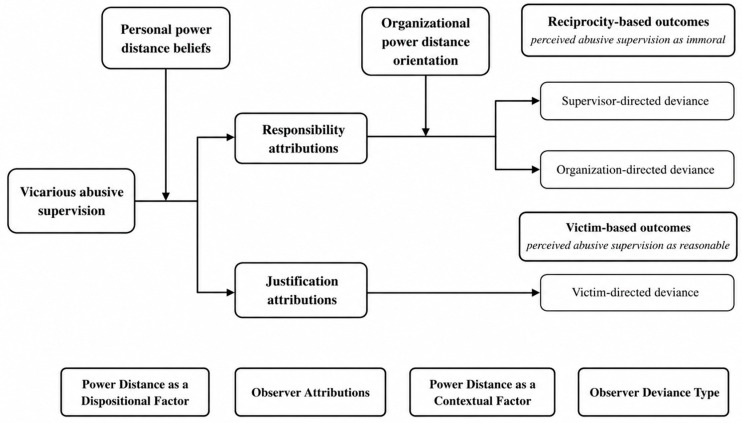
Proposed integrative model of deviant observer behaviors in response to vicarious abusive supervision, incorporating the mediating effect of attribution directions and the moderating effects of personal/organizational-level power distance orientations.

To avoid confusion at the level of analysis^1^, we clarify that, in this work, attributions are discussed as event-level cognitions. Regardless of trait-based attribution styles (e.g., hostile attribution style; Harvey & Martinko, 2009), we explore employees’ causal explanations for observed abusive supervision (i.e., an event-specific behavior) in the workplace. Furthermore, we consider the role of personal power distance orientation (i.e., a personal-level trait) in predicting these evaluations. In addition, to argue the organization’s impact within this process, we include organizational power distance as a contextual variable. Figure 1 shows the proposed conceptual model.

### 3.1. Reciprocity-Based Outcomes and Responsibility Attributions

When addressing reciprocity-based outcomes, we refer to observer deviant behavior directed at an agent with a desire to act against unfairness. These actions are motivated by purposes such as punishment, revenge, or retribution. This means that the abusive behaviors are perceived as inappropriate, and the observer blames a target for the action. Responsibility or blame attributions are morally based judgments that focus more on aversive or negative outcomes (Mikula, 1993; Malle, 2007). Causality is attributed to the agent whose intentionality engaged the action (Malle et al., 2014) and who has the ability to control it (Coeckelbergh, 2020). Weiner’s (1979) three-dimensional model argues that there are three fundamental dimensions to consider when trying to understand the cause of an action: the actor’s relationship to the action (i.e., locus), the duration of the cause (i.e., stability), and the actor’s power over the action (i.e., control). That is, if the abuse occurs at the abuser’s own initiative, is not a one-time event, and the abuser chooses to act in accordance, then the blame is attributed to the agent. Weiner (1985) also emphasized that causal dimensions (e.g., intentionality) can trigger various emotions (e.g., anger, pity) and that these emotions can, in turn, lead to behavioral consequences. In this regard, the perceived stability or the intentional nature of the abuse may reveal aggression triggered by feelings of anger, as shown in previous studies (e.g., Mitchell et al., 2015; Zhang et al., 2020).

In this regard, the first target could be the supervisor who committed the action. The observer may evaluate abuse as a supervisor’s intentional (i.e., dispositional; Jones & Davis, 1965) behavior, and performed by his/her own free will. While there are circumstances in which the actor is completely free to choose his action, there are other factors (e.g., the environment; Jones & Davis, 1965) that may shape observers’ judgments. For example, external influences (e.g., the organization) may affect observers’ perceptions of the perpetrator’s responsibility (see Section 3.4 for a more detailed discussion). Whether or not these factors are present, if the supervisor who commits the abuse is perceived as responsible, it may lead to behaviors directed toward the supervisor.

Although third-party research shows that (Mitchell et al., 2015; Zhang et al., 2020) observers of vicarious abusive supervision can develop aggression toward supervisors, empirical findings on the relationship between this and observer attributions are still limited. But we have a few studies that can be given as examples. For example, Chen and Liu (2019) found that when bystanders attributed stronger responsibility for abusive supervision to the supervisor (i.e., supervisor-directed attribution), they exhibited higher levels of deviance behaviors towards the supervisor (i.e., supervisor-directed deviance). Given that, we propose that blaming the supervisor among observers who perceive abusive supervision as an inappropriate behavior may lead to aggression against the supervisor.

**Proposition** **1.** *When holding justification attribution constant, observers’ responsibility attributions for abusive supervision will be positively associated with the frequency and severity of supervisor-directed deviant behaviors.*

The context in which the abusive supervision takes place may also have an impact on observer judgments. Bowling and Beehr (2006) claim that responsibility can also be attributed to the organization as a result of different factors, such as organizational climate. As previously stated, causality is attributed to the agent who has control over the action. Evaluating from Weiner’s (1979) perspective, the organization that has the power (i.e., control) to terminate the abuse, by not choosing to stop it, may become another target of causality. In an organizational context, the ultimate power that can influence the supervisor is the organization itself, as it is the only force that can constrain and reduce the power of the powerful individuals within its system (Keltner et al., 2003). The organization’s perceived responsibility for the continuity of the process may, by triggering anger (Weiner, 1985), shift the focus of the attack to the organization. Moreover, the power that supervisors have is derived from their position and the formal structure (O’reilly & Aquino, 2011) and is given to them by the organization. Therefore, the organization may be perceived as a facilitator and found responsible for giving the authority to the abusive supervisor. Furthermore, the decline in perceived organizational justice resulting from abusive supervision (Tepper, 2000) may also lead the organization to be a target of the observer’s morally based judgments.

As Dhanani and LaPalme (2019) underline, as a result of mistreatment, observers can display antisocial behavior not only against the perpetrator but also against the organization itself. Despite studies (e.g., Mitchell & Ambrose, 2007) explaining organization-directed aggression as a form of displaced aggression, Tepper et al. (2008) propose that these behaviors may emerge when the organization is held responsible for abusive supervisors. In their study, which drew on workplace attributions, Bowling and Michel (2011) showed that when the organization is held responsible for abuse, hostile behaviors towards the organization may develop. On the other hand, the findings of a case study examining bystander perspective observers of workplace mistreatment (i.e., bullying) may engage in counterproductive behaviors toward the organization if they believe the organization is unable to prevent this mistreatment (Wu & Wu, 2018). Considering all this information, we propose that observers may also exhibit deviant behavior directed at the organization due to vicarious abuse if they make blame attributions.

**Proposition** **2.** *When holding justification attribution constant, observers’ responsibility attributions for abusive supervision will be positively associated with the frequency and severity of organization-directed deviant behaviors.*

Taken together, responsibility judgments imply that the incident is perceived as undesirable and that a target is perceived as guilty for the act. As perceived responsibility for an unwanted event gives rise to emotions like anger (Weiner, 1985, 2007), similar to deontic actions, the fundamental motive underlying observers’ deviant responses is to act for justice and to *stand by* the victim. However, as noted earlier, there are also situations in which observer aggression is directed *against* the victim.

### 3.2. Victim-Based Outcomes and Justification Attributions

So, why do some observers seek revenge against the perpetrator, and some of them become one? When addressing victim-based outcomes, we refer to observer deviant behavior directed at the victim. According to Mikula (2003), moral violations may not always trigger a sense of injustice. The existence of sufficient justifications (i.e., reasonable explanations for intentional events; Malle et al., 2014) of a particular action may affect judgments of moral violation and play a central role in assessing an action (Mikula, 1993, 2003). That is, perceived reasonability can justify a violent event, which may lead to these behaviors being seen as appropriate behaviors in the workplace—and even imitated. Empirical findings support the idea that while observers are evaluating abusive behavior, through different mechanisms, they may not see the act as a complete moral violation. These reasons may serve as explanations that justify the action and make it seem legitimate.

Before conceptualizing justification attribution, it would be appropriate to mention several key approaches that are widely used while justifying a moral violation. As discussed below, some observers may believe that the victim deserved the abuse. This perception shows similarity to the notion of just-world beliefs—the idea that everyone gets what they deserve (Lerner, 1980; Furnham, 2003). Due to their various ideologies (such as respect for authority), some observers may not consider an abuse that is used to preserve and defend the social order to be unjust, reflecting system justification (Jost & Liviatan, 2007) efforts. Another concept closely related to justifications is moral disengagement. The theory of moral disengagement (Bandura et al., 1996; Moore, 2015) posits that people use various cognitive mechanisms (e.g., moral justification) to help themselves justify an unethical action that does not align with their internal moral standards. Moral disengagement mechanisms can be viewed as cognitive efforts to reframe a person’s behavior (Moore, 2015; Moore et al., 2012) in order to preserve self-respect. Justification attributions, on the other hand, reflect an attempt to evaluate a third-party’s (e.g., supervisor) behavior under situational circumstances (e.g., abusive supervision). While moral disengagement efforts are personal-level mechanisms for reframing, justification attributions refer to event-level, related evaluation efforts. Consequently, the concepts of “justification attributions” that we propose here and “moral disengagement mechanisms” refer to different concepts, although they may be related to similar cognitive processes. It can be seen that cognitive evaluations of a moral violation might be explained through various mechanisms and different theoretical perspectives. The term we conceptualize below as “justification attributions” should be viewed as an overarching framework that encompasses all these mechanisms. The distinctive feature of our attribution-based perspective is that it approaches justifications as a mediator, focusing on the observers’ attribution direction rather than the efforts or reasons they can use while justifying an immoral action.

As discussed above, justifications can be influenced by various factors. For example, stereotypes (e.g., laziness) can serve as explanations about unwanted events to rationalize and legitimate the authority (Jost & Liviatan, 2007). For example, Xiang et al. (2026) found that the social relationship between the violator and the observer reduced third-party norm violation punishment. P. Liu et al. (2022) found that observed leader incivility could lead observers to define the target employee as an organizational *outsider*, which in turn may lead them to engage in deviant behavior towards the victim. In their study, Yu et al. (2022) found that observers who feel content about the abuse have the belief that the victim *deserves* the mistreatment. Chen and Liu (2019) found that observers’ perception of the relationship with supervisors (i.e., leader-member exchange) and their identification with the manager weakened the relationship between indirect abusive supervision and attributions directed towards the supervisor, due to the group’s internal perception. Xu et al. (2020) reported that observers could engage in deviant behavior towards the abused victim when they perceived the victim as a *rival*. Again, from Weiner’s (1985) perspective, all these interpretations can be related to the observer’s satisfaction with the action, which can also affect observer response.

Taking all of these studies into account, we conceptualize justifications attributions as an observer attribution who perceives abusive behaviors as reasonable and justifies the abuse via various explanations. Harvey et al. (2014) suggest that an employee’s attributions can explain her/his decision to engage in a behavior which may be seen as unjust by others. Therefore, we also argue that this type of attribution may serve as an antecedent to mistreatment directed to the victim, and we propose the following:

**Proposition** **3.** *When holding responsibility attribution constant, observers’ justification attributions for abusive supervision will be positively associated with the frequency and severity of victim-directed deviant behaviors*.

### 3.3. A Dispositional Factor: Observer’s Power Distance Beliefs

As clarifying the boundaries is a valuable effort in theory building (Colquitt & Zapata-Phelan, 2007), we are adding power distance as a dispositional factor to our model. Power distance orientation is a cultural value (Hofstede, 1983; Hofstede et al., 2005) that is frequently studied in the field of organizational psychology, particularly in the context of the superior-subordinate relationship. While initial studies focus on national-level analysis, because cultural values can also reveal cultural variation within a single country (Kirkman et al., 2009), the proper approach is to analyze the effects of power distance beliefs at the individual level. Therefore, we add observers’ values regarding the distribution of power in our model as a dispositional factor.

As discussed earlier, the attribution process is a reasoning process of evaluating (prior) information and beliefs (Kelley & Michela, 1980). Personal power distance beliefs refer to personal beliefs about power, authority, hierarchy, and status (Kirkman et al., 2009). People with a high-power distance orientation respect authority. They view power differences between people as legitimate and even believe that those in power may have certain privileges. People with a low power distance, on the other hand, are more egalitarian. Subordinates with lower power distance are more likely to question and/or challenge the upper-level positions, while subordinates with higher power distance are more open to obeying their leaders (Carl et al., 2004). Empirical findings suggest that power distance can influence organizational outcomes. For example, W. Wang et al. (2012) found that employees who had lower power distance beliefs perceived more interactional injustice about abusive supervision, and because of that, they engaged in more deviant behaviors than those who had higher levels of power distance.

That is, an immoral act can be interpreted differently—or even justified—depending on one’s beliefs about the behavior of someone who is an authority figure. In other words, an abusive behavior may be justified through a reasonable belief that the “supervisor has the right to do so.” As the perceiver’s ideology has an impact on reducing the blame through justification (Tetlock et al., 2007; Malle et al., 2014) and power distance is an ideology that helps members maintain the status quo (Jost & Liviatan, 2007), we propose:

**Proposition** **4.** *Personal power distance beliefs moderate the relationship between vicarious abusive supervision and observer attributions. Specifically, higher power distance beliefs will (a) weaken the positive relationship between vicarious abusive supervision and responsibility attributions and (b) strengthen the positive relationship between vicarious abusive supervision and justification attributions.*

### 3.4. A Contextual Factor: The Organization’s Power Distance Orientation

As discussed above, organizational factors may affect an employee’s evaluation of workplace harassment, which may lead to finding the organization partially or directly guilty and resulting in organizational deviance (Bowling & Beehr, 2006). Because understanding contextual factors is very important in workplace mistreatment (Hershcovis et al., 2020), we explore the organization’s culture role in vicarious abusive supervision by adding organizational power distance to our conceptualized model. Thus, we provide a contextual factor that may have an impact on observer evaluations.

Organizational power distance refers to the organizational norms regarding the centralization of authority and hierarchy. Although differences in power and authority are a natural aspect of organizational structures (Yang et al., 2007), the distribution of power reveals inequalities at different levels. In low power distance organizations, equality rather than hierarchical position is at the forefront, while in high power distance organizations, showing obedience and respect to authority figures (i.e., superiors) is expected (T. N. Ma et al., 2025). In organizations where power is not equally distributed, the power of superiors is elevated (Carl et al., 2004), and there are strict rules such as loyalty to the superior.

As high-power-distance practices raise the supervisor’s authority (Carl et al., 2004), in this type of organizational structure, employees perceived organizational support may decrease because the perception that the organization supports the supervisor—in our case, the perpetrator—is likely to be higher. Therefore, the organization can be perceived as a facilitator of abuse and can be seen as one of the parties responsible for it. And as discussed earlier, this could lead to retaliatory actions against the organization (e.g., Tepper et al., 2008; Bowling & Michel, 2011). Taking all of this into account, we argue that retaliatory behaviors toward the organization will increase among observers who make blame attributions as the organization’s power distance beliefs rise. Therefore, we propose:

**Proposition** **5.** *The organization’s power distance norms will moderate the relationship between responsibility attributions and reciprocity-based outcomes. Specifically, higher organizational power distance norms will strengthen the positive relationship between responsibility attributions and organization-directed deviance.*

In addition, as employees with high perceptions of power distance view power differences as legitimate and they respect hierarchy (Kirkman et al., 2009), they are more likely to show obedience (T. N. Ma et al., 2025) to the organizational formal structure expected of them. Whereas employees with lower power distance beliefs are more open to challenging organizational practices (Carl et al., 2004). As a result, in a higher organizational power distance environment, observers’ personal power distance orientation may influence deviant responses toward the organization due to their belief in challenging authority. Therefore, we propose:

**Proposition** **6.** *Within a higher organizational power distance context, the positive relationship between responsibility attributions and organization-directed deviance should be stronger for observers with lower power distance beliefs.*

## 4. Discussion

According to Bowling and Beehr (2006), research on workplace harassment tends to take a black-box approach while explaining the potential reasons (e.g., the attribution process) for the relationships between antecedents and consequences. However, identifying the underlying causes of expected relationships is a key step toward a better understanding of organizational phenomena. To understand the observer deviance resulting from abusive supervision—which has mostly been examined from an emotional perspective—we focused on the observers’ cognitive processes. Our work advances both vicarious abusive supervision and workplace attribution research by offering two potential attribution directions leading to bystander aggression. Moreover, our conceptual approach allows us to provide a possible answer to explaining observer mistreatment of victims, which is inconsistent with deontic theory.

### 4.1. Theoretical Implication

This paper makes several important theoretical contributions. First, we propose a different perspective from deontic justice, which is one of the most common approaches that examines observers’ behaviors. Dhanani and LaPalme (2019) pointed out that vicarious mistreatment increases overall levels of negative behavior, which could also involve behavior (e.g., towards the victim) that cannot be explained via a deontic perspective. Again, Hill and colleagues (2025) pointed out that an observer’s pleasure from the mistreatment is not consistent with a deontic desire. Therefore, we shifted our focus from an emotion-centered approach to a cognition-centered approach in the observer’s perception of abuse. However, it is important to note that cognitive processes cannot be completely separated from emotional processes. Lazarus (1991) states that emotions may be the result of subjective evaluations of events. Although there are conflicting viewpoints on which one takes precedence (e.g., cognition as a determinant of emotions: Weiner et al., 1988; affective states as an information source: Schwarz & Clore, 1983), these two personal variables may be in interaction. On the other hand, as previously emphasized, this work considers the possibility that abusive behaviors may not always be viewed as harmful or may be perceived as reasonable by an observer. Through this, as attributions can explain an employee’s behavior that may be seen as unjust (Harvey et al., 2014), they offer an alternative explanation for the observer’s harmful behavior directed at the victim. Consequently, in its current form, this work can be viewed as an effort to provide an integrated model of antisocial observer outcomes, including deviance directed at the victim. More research is needed to improve our understanding of observer evaluations about vicarious mistreatment.

Second, we advance the workplace attribution research by incorporating a third-party perspective. As Martinko et al. (2011) pointed out, although it has not been given the importance it deserves in organizational science, attribution theory can be an important tool for us due to its explanatory power. Similar to the efforts (e.g., taking the perspective of the perpetrator; Fiori et al., 2016) that use attribution theory to explain why observers try to justify the perpetrator’s actions, we argue that observers may not always tend to blame the perpetrator. As discussed in detail above, responsibility attributions can be seen as a process to blame the perpetrator or the organization. By its very nature, the attribution of responsibility is based on the tendency to view the act as unwanted or morally false. The actor, who intentionally committed abusive actions (Jones & Davis, 1965; Malle et al., 2014) and had the ability to control them (Weiner, 1979), was blamed by an observer. The observer is concerned by the action and, in accordance with the norm of reciprocity, may feel the need to respond. In contrast, justification attributions reflect efforts to legitimate the moral violation. As we proposed, it is more of an attempt to justify the event, absolve the target’s responsibility, and distance the act from intentionality by various explanations. By offering justification attributions as another type of attribution that observers can make, we present a cognition-based explanation that may result in victim-directed mistreatment, which has been mostly explained through emotions (e.g., schadenfreude). Mikula (2003) found that justice judgments were affected by justifications. In many studies (e.g., Ng et al., 2020; P. Liu et al., 2021; Yu et al., 2022), the observers’ perspectives about the actors (e.g., the victim “deserving” it) is one of the key factors in the assessment of mistreatment directed at the victim and, consequently, in the continuation of violence against the victim. Such thoughts can play an important role in evaluating an immoral event and may result in justifying or accepting the abuse. System justification refers to a psychological tendency to justify and defend the status quo and prefer the existing social arrangement even if this is against the societal interests (Jost & Liviatan, 2007). In an environment where mistreatment can be tolerated, it will not be surprising to see it spread throughout the organization. That is, justifications can serve as a contributor in reinforcing and sustaining a negative organizational climate. For this reason, we argue that justification attributions may be a valuable variable to examine in an organizational context.

Although personal characteristics (e.g., conscientiousness) were examined as antecedents of attributions (see Bowling & Beehr, 2006), personal values (e.g., power distance beliefs) were neglected. Following Breaux et al. (2010)’s suggestions to search for the information employees use for attribution, we add individual power distance orientation to our model. Individuals with high power distances may respect their authority figures, and rarely question their actions (Carl et al., 2004), therefore, they may be more likely to follow their superiors. For example, Bai et al. (2022) emphasized that an observer’s mistreatment of a victim may be intended as a gesture to please their superior. Therefore, we argue that—particularly given their views on the status quo—the observer’s power distance beliefs can be seen as a significant employee value that may influence the judgment of abuse and, more importantly, the justification of it.

Finally, taking into account the impact of the organizational climate on employee perceptions, we discuss the potential effects of organizational power distance, which is a dimension of organizational culture. Kelley and Michela (1980) emphasized the importance of analyzing the context in which the attribution took place. Bowling and Beehr (2006) state that the created organizational culture or human research practices can be seen as reasons to attribute the organization. In the workplace, superior-subordinate relationships are closely linked to organizational norms regarding the distribution of power. For this reason, we believe organizational power distance is a worthwhile factor for further examination, particularly in the workplace mistreatment context.

### 4.2. Practical Implications

Bystander attitudes are a key factor in whether negative managerial behavior persists or ceases. However, the organization itself, not a coworker, should be responsible for destructive managerial behaviors. Therefore, we believe it is important for the organization to take the necessary steps to prevent bystander aggression and demonstrate that it supports its employees. Employees who found the organization responsible for the abuse may tend to think that the organization created this negative climate (see Proposition 5). Therefore, in an environment where negative behaviors are thought to be accepted and normative, observers may be more likely to engage in antisocial behavior against more and different targets. Ensuring a fair organizational climate and implementing organizational policies to prevent abuse could help restrain the spread of negative workplace behavior through a trickle-down effect. For this reason, it is important to once again emphasize the importance of creating a more supportive and egalitarian organizational culture. Training sessions on the organizational power, power dynamics in organizational processes, and the contextual implications of personal judgments regarding distribution of power (see Proposition 4) will also provide valuable insights. Although this paper focuses on third-party retaliatory behaviors, there are also prosocial aspects to supporting victims. Therefore, firstly it is important to train staff on prosocial and antisocial behavior patterns. In addition to raising awareness, we believe that training programs designed to address the steps for third-party employees who observe a toxic management style could help. These educational programs can guide employees to ensure the process is conducted effectively, without being hostile toward the organization (see Proposition 2) or the abuser (see Proposition 1). Unfortunately, not every victim may receive coworker support. Yu et al. (2022) reported that some of the observers of vicarious abusive supervision were scared and motivated to act “especially careful” in order not to face the same situation one day. However, displaying aggression toward the victim (see Proposition 3) cannot be an excuse for this fear. A coworker’s victimizing of the victim may lead other coworkers to mistreat the victim as well, and turn this employee into a kind of organizational target. Therefore, we believe that training interventions regarding abuser-supporters, and victim-blaming may provide employees with valuable insights to other coworkers in the process.

### 4.3. Limitations and Future Directions

Our work has certain limitations. First, research is warranted to provide empirical evidence in support of our proposed model. While there are studies (e.g., Bowling & Michel, 2011; Breaux et al., 2010) that use scales to measure blame attributions regarding abusive supervision, to the best of our knowledge, there is no validated scale that specifically addresses justifications when assessing supervisor abuse. Therefore, a justification attribution scale should first be developed for the observed abusive supervision. Bandura et al.’s (1996) or Moore et al.’s (2012) scales on moral disengagement could serve as a good starting point for efforts to establish the scale. However, there are certain factors to consider when developing the scale. First of all, existing measure items and dimensions should be evaluated with great care in the scale development process. Although the terms used in the scales could be similar to the “justification attribution” provided in this paper, the concepts could be entirely different. For example, Bandura et al.’s (1996) Mechanisms of Moral Disengagement Scale for moral justification dimension, involves justifying a person’s engagement in immoral actions in order to serve a morally better cause (e.g., “It is alright to beat someone who bad mouths your family”; Bandura et al., 1996, p. 374). Similarly, Moore et al.’s (2012) moral justification dimension item in the Propensity to Morally Disengage Scale does not reflect our construct (e.g., “It is okay to spread rumors to defend those you care about”; Moore et al., 2012, p. 47). Whereas attribution of blame dimensions of both studies has more appropriate items to adapt to our construct (e.g., “Kids who get mistreated usually do things that deserve it” (Bandura et al., 1996, p. 374); “People who get mistreated have usually done something to bring it on themselves” (Moore et al., 2012, p. 48). However, these items do not reflect any attempts to provide an employee’s justification of managerial mistreatment in a workplace context. An example of a scale item that could be adapted by these measures and abusive supervision scale (Mitchell & Ambrose, 2007; Tepper, 2000) might be: “My coworker who is told by our supervisor that s/he is incompetent, usually does things to deserve it”. Nevertheless, the developed scale items have to demonstrate discriminant validity, to show the novelty of the developed measure and to put forward the distinct structure of these two concepts (Churchill, 1979; Boateng et al., 2018). Therefore, future research should verify, through assessment methods (e.g., the multitrait–multimethod matrix, the heterotrait–monotrait ratio, and confirmatory factor analysis), that justification attribution constitutes a latent construct distinct from moral disengagement. Before developing items, it is also recommended to draw on findings from qualitative research (e.g., “He (the victim) was wrong. He didn’t deserve sympathy, and the supervisor just did the right thing”; Yu et al., 2022, p. 4).

Second, because of the empirical findings reviewed in this work, the model focuses exclusively on abusive supervision. A more comprehensive model could include other negative managerial behaviors (e.g., bullying, mobbing, toxic leadership) or different antisocial observer behaviors. Moreover, as the observers’ aggression towards the victim is a relatively new area of research, studies on this topic are also limited. We need more research on bystander aggression directed at victims. Not supporting the victim may be understandable due to a fear of becoming the next one. However, understanding the justification—and even imitating—abuse can be seen as prior knowledge on the emergence process of future abusers. Additionally, peer-directed deviance can be added to the model as another form of observer aggression. If observers can engage in victim-directed deviance, they can certainly direct it to another coworker as well. A final recommendation for future research is including additional observer values. For example, collective people give greater importance to group values (Peng et al., 2001) and may tend to see their peers as a group member. This may result in them making stronger attributions of blame or being more likely to engage in reciprocity-based reactions.

## 5. Conclusions

In this paper, we use attribution theory to explore why observers of abusive supervision may not always act in accordance with deontic responses to their coworker’s mistreatment. Furthermore, we discuss the role of observers’ power distance beliefs and the organization’s power distance norms in the evaluation process of supervisor abuse. By presenting a potential cognitive reasoning process underlying negative bystander outcomes, we aim to offer new insights for future research on bystander responses to abusive supervision, which has been mostly examined from an emotion-centered perspective. We hope our work contributes to scholars giving more attention to workplace attribution research.

## Data Availability

No new data were created or analyzed in this study. Data sharing is not applicable to this article.

## Note

1 While empirically testing the model, whereas personal power distance beliefs can be measured directly, it would be more appropriate to employ a research design (e.g., a between-subjects design) that enables vignette-level measurement of observed abusive supervision, as it is an event-level variable.

## References

[B1-behavsci-16-01191] Bai Y., Lu L., Lin-Schilstra L. (2022). Auxiliaries to abusive supervisors: The spillover effects of peer mistreatment on employee performance. Journal of Business Ethics.

[B2-behavsci-16-01191] Bandura A. (1977). Self-efficacy: Toward a unifying theory of behavioral change. Psychological Review.

[B3-behavsci-16-01191] Bandura A., Barbaranelli C., Caprara G. V., Pastorelli C. (1996). Mechanisms of moral disengagement in the exercise of moral agency. Journal of Personality and Social Psychology.

[B4-behavsci-16-01191] Bennett R. J., Robinson S. L. (2000). Development of a measure of workplace deviance. Journal of Applied Psychology.

[B5-behavsci-16-01191] Boateng G. O., Neilands T. B., Frongillo E. A., Melgar-Quiñonez H. R., Young S. L. (2018). Best practices for developing and validating scales for health, social, and behavioral research: A primer. Frontiers in Public Health.

[B6-behavsci-16-01191] Bowling N. A., Beehr T. A. (2006). Workplace harassment from the victim’s perspective: A theoretical model and meta-analysis. Journal of Applied Psychology.

[B7-behavsci-16-01191] Bowling N. A., Michel J. S. (2011). Why do you treat me badly? The role of attributions regarding the cause of abuse in subordinates’ responses to abusive supervision. Work & Stress.

[B8-behavsci-16-01191] Breaux D. M., Tepper B. J., Carr J. C., Folger R. G. (2010). An attributional analysis of employees’ responses to abusive supervision. The “dark” side of management.

[B9-behavsci-16-01191] Carl D., Gupta V., Javidan M., House R. J., Hanges P. J., Javidan M., Dorfman P. W., Gupta V. (2004). Power distance. Culture, leadership and organizations: The GLOBE study of 62 societies.

[B10-behavsci-16-01191] Chen S. C., Liu N. T. (2019). When and how vicarious abusive supervision leads to bystanders’ supervisor-directed deviance: A moderated–mediation model. Personnel Review.

[B11-behavsci-16-01191] Churchill G. A. (1979). A paradigm for developing better measures of marketing constructs. Journal of Marketing Research.

[B12-behavsci-16-01191] Coeckelbergh M. (2020). Artificial intelligence, responsibility attribution, and a relational justification of explainability. Science and Engineering Ethics.

[B13-behavsci-16-01191] Colquitt J. A., Zapata-Phelan C. P. (2007). Trends in theory building and theory testing: A five-decade study of the Academy of Management Journal. Academy of Management Journal.

[B14-behavsci-16-01191] Cropanzano R., Goldman B., Folger R. (2003). Deontic justice: The role of moral principles in workplace fairness. Journal of Organizational Behavior.

[B15-behavsci-16-01191] Dhanani L. Y., LaPalme M. L. (2019). It’s not personal: A review and theoretical integration of research on vicarious workplace mistreatment. Journal of Management.

[B16-behavsci-16-01191] Di Stefano G., Scrima F., Parry E. (2019). The effect of organizational culture on deviant behaviors in the workplace. The International Journal of Human Resource Management.

[B17-behavsci-16-01191] Fiori M., Krings F., Kleinlogel E., Reich T. (2016). Whose side are you on? Exploring the role of perspective taking on third-party’s reactions to workplace deviance. Basic and Applied Social Psychology.

[B18-behavsci-16-01191] Folger R., Gilliland S., Steiner D., Skarlicki D. (2001). Fairness as deonance. Theoretical and cultural perspectives on organizational justice.

[B19-behavsci-16-01191] Furnham A. (2003). Belief in a just world: Research progress over the past decade. Personality and Individual Differences.

[B20-behavsci-16-01191] Gouldner A. W. (1960). The norm of reciprocity: A preliminary statement. American Sociological Review.

[B21-behavsci-16-01191] Harvey P., Madison K., Martinko M., Crook T. R., Crook T. A. (2014). Attribution theory in the organizational sciences: The road traveled and the path ahead. Academy of Management Perspectives.

[B22-behavsci-16-01191] Harvey P., Martinko M. J., Borkowski N. (2009). Chapter 7. Attribution theory and motivation. Organizational behavior, theory and design in healthcare.

[B23-behavsci-16-01191] Hershcovis M. S., Cortina L. M., Robinson S. L. (2020). Social and situational dynamics surrounding workplace mistreatment: Context matters. Journal of Organizational Behavior.

[B24-behavsci-16-01191] Hill E. T., Colquitt J. A., Burgess R., Priesemuth M., McClain J. T. (2025). Third-party perceptions of mistreatment: A meta-analysis and integrative model of reactions to perpetrators and victims. Journal of Applied Psychology.

[B25-behavsci-16-01191] Hofstede G. (1983). The cultural relativity of organizational practices and theories. Journal of International Business Studies.

[B26-behavsci-16-01191] Hofstede G., Hofstede G. J., Minkov M. (2005). Cultures and organizations: Software of the mind.

[B27-behavsci-16-01191] Jones E. E., Davis K. E. (1965). From acts to dispositions the attribution process in person perception. Advances in experimental social psychology.

[B28-behavsci-16-01191] Jost J. T., Liviatan I., Baumeister R. F., Vohs K. D. (2007). System justification. Encyclopedia of social psychology.

[B29-behavsci-16-01191] Kelley H. H. (1973). The processes of causal attribution. American Psychologist.

[B30-behavsci-16-01191] Kelley H. H., Michela J. L. (1980). Attribution theory and research. Annual Review of Psychology.

[B31-behavsci-16-01191] Keltner D., Gruenfeld D. H., Anderson C. (2003). Power, approach, and inhibition. Psychological Review.

[B32-behavsci-16-01191] Kirkman B. L., Chen G., Farh J. L., Chen Z. X., Lowe K. B. (2009). Individual power distance orientation and follower reactions to transformational leaders: A cross-level, cross-cultural examination. Academy of Management Journal.

[B33-behavsci-16-01191] Lazarus R. S. (1991). Progress on a cognitive-motivational-relational theory of emotion. American Psychologist.

[B34-behavsci-16-01191] Lerner M. J. (1980). The belief in a just world. The belief in a just world: A fundamental delusion.

[B35-behavsci-16-01191] Lian H., Brown D. J., Ferris D. L., Liang L. H., Keeping L. M., Morrison R. (2014). Abusive supervision and retaliation: A self-control framework. Academy of Management Journal.

[B36-behavsci-16-01191] Lian H., Ferris D. L., Brown D. J. (2012). Does power distance exacerbate or mitigate the effects of abusive supervision? It depends on the outcome. Journal of Applied Psychology.

[B37-behavsci-16-01191] Liu C., Yang J., Liu J., Zhu L. (2021). The effect of abusive supervision on employee deviant behaviors: An identity-based perspective. The International Journal of Human Resource Management.

[B38-behavsci-16-01191] Liu P., An X., Li X. (2022). You are an outsider! How and when observed leader incivility affect hospitality employees’ social categorization and deviant behavior. International Journal of Hospitality Management.

[B39-behavsci-16-01191] Liu P., Li X., Li A., Wang X., Xiong G. (2021). How third parties respond to workplace incivility: The moderating role of the dark triad and task interdependence. Personality and Individual Differences.

[B40-behavsci-16-01191] Lunenburg F. C. (2012). Power and leadership: An influence process. International Journal of Management, Business, and Administration.

[B41-behavsci-16-01191] Ma B., Tillman C. J., Pan J. (2017). Third party judgment and reaction to abusive supervision of coworkers. Academy of management proceedings.

[B42-behavsci-16-01191] Ma T. N., Yeh Y. J. Y., Lee H. Y., Vu H. V. (2025). The impact of customer incivility on employee negative emotions: An organizational culture perspective. Management Research Review.

[B43-behavsci-16-01191] Malle B. F., Baumeister R. F., Vohs K. D. (2007). Attributions. Encyclopedia of social psychology.

[B44-behavsci-16-01191] Malle B. F., Guglielmo S., Monroe A. E. (2014). A theory of blame. Psychological Inquiry.

[B45-behavsci-16-01191] Martinko M. J., Harvey P., Dasborough M. T. (2011). Attribution theory in the organizational sciences: A case of unrealized potential. Journal of Organizational Behavior.

[B46-behavsci-16-01191] Mikula G. (1993). On the experience of injustice. European Review of Social Psychology.

[B47-behavsci-16-01191] Mikula G. (2003). Testing an attribution-of-blame model of judgments of injustice. European Journal of Social Psychology.

[B48-behavsci-16-01191] Mitchell M. S., Ambrose M. L. (2007). Abusive supervision and workplace deviance and the moderating effects of negative reciprocity beliefs. Journal of Applied Psychology.

[B49-behavsci-16-01191] Mitchell M. S., Vogel R. M., Folger R. (2015). Third parties’ reactions to the abusive supervision of coworkers. Journal of Applied Psychology.

[B50-behavsci-16-01191] Moore C. (2015). Moral disengagement. Current Opinion in Psychology.

[B51-behavsci-16-01191] Moore C., Detert J. R., Klebe Treviño L., Baker V. L., Mayer D. M. (2012). Why employees do bad things: Moral disengagement and unethical organizational behavior. Personnel Psychology.

[B52-behavsci-16-01191] Ng K., Niven K., Hoel H. (2020). ‘I could help, but…’: A dynamic sensemaking model of workplace bullying bystanders. Human Relations.

[B53-behavsci-16-01191] O’reilly J., Aquino K. (2011). A model of third parties’ morally motivated responses to mistreatment in organizations. Academy of Management Review.

[B54-behavsci-16-01191] Peng K., Ames D. R., Knowles E. D., Matsumoto D. (2001). Culture and human inference: Perspectives from three traditions. The handbook of culture and psychology.

[B55-behavsci-16-01191] Priesemuth M. (2013). Stand up and speak up: Employees’ prosocial reactions to observed abusive supervision. Business & Society.

[B56-behavsci-16-01191] Robinson S., Bennett R. (1995). A typology of deviant workplace behaviors: A multidimensional scaling study. Academy of Management Journal.

[B57-behavsci-16-01191] Schwarz N., Clore G. L. (1983). Mood, misattribution, and judgments of well-being: Informative and directive functions of affective states. Journal of Personality and Social Psychology.

[B58-behavsci-16-01191] Shao Q., Zhang K., Zhao X. (2026). An intuitive model of bystander responses to workplace mistreatment. Behavioral Sciences.

[B59-behavsci-16-01191] Shoss M. K., Eisenberger R., Restubog S. L. D., Zagenczyk T. J. (2013). Blaming the organization for abusive supervision: The roles of perceived organizational support and supervisor’s organizational embodiment. Journal of Applied Psychology.

[B60-behavsci-16-01191] Skarlicki D. P. (2001). Cross-cultural perspectives of organizational justice. International Journal of Conflict Management.

[B61-behavsci-16-01191] Skarlicki D. P., Rupp D. E. (2010). Dual processing and organizational justice: The role of rational versus experiential processing in third-party reactions to workplace mistreatment. Journal of Applied Psychology.

[B62-behavsci-16-01191] Sulea C., Fine S., Fischmann G., Sava F. A., Dumitru C. (2013). Abusive supervision and counterproductive work behaviors: The moderating effects of personality. Journal of Personnel Psychology.

[B63-behavsci-16-01191] Tepper B. J. (2000). Consequences of abusive supervision. Academy of Management Journal.

[B64-behavsci-16-01191] Tepper B. J., Henle C. A., Lambert L. S., Giacalone R. A., Duffy M. K. (2008). Abusive supervision and subordinates’ organization deviance. Journal of Applied Psychology.

[B65-behavsci-16-01191] Tetlock P. E., Visser P. S., Singh R., Polifroni M., Scott A., Elson S. B., Mazzocco P., Rescober P. (2007). People as intuitive prosecutors: The impact of social-control goals on attributions of responsibility. Journal of Experimental Social Psychology.

[B66-behavsci-16-01191] van Dijk W. W., Ouwerkerk J. W. (2014). Introduction to schadenfreude. Schadenfreude: Understanding pleasure at the misfortune of others.

[B67-behavsci-16-01191] Wang H., Xiao J. (2021). Examining the within-person effects of abusive supervision on multifoci deviance: Ethical climate as a moderator. Business Ethics, the Environment & Responsibility.

[B68-behavsci-16-01191] Wang W., Mao J., Wu W., Liu J. (2012). Abusive supervision and workplace deviance: The mediating role of interactional justice and the moderating role of power distance. Asia Pacific Journal of Human Resources.

[B69-behavsci-16-01191] Wei W., Chen H., Feng J., Li J. (2023). Helpful or hurtful? A study on the behavior choice of bystanders in the context of abusive supervision. International Journal of Conflict Management.

[B70-behavsci-16-01191] Weiner B. (1979). A theory of motivation for some classroom experiences. Journal of Educational Psychology.

[B71-behavsci-16-01191] Weiner B. (1985). An attributional theory of achievement motivation and emotion. Psychological Review.

[B72-behavsci-16-01191] Weiner B., Baumeister R. F., Vohs K. D. (2007). Responsibility attributions. Encyclopedia of social psychology.

[B73-behavsci-16-01191] Weiner B., Perry R. P., Magnusson J. (1988). An attributional analysis of reactions to stigmas. Journal of Personality and Social Psychology.

[B74-behavsci-16-01191] Wu S. H., Wu C. C. (2018). Bullying bystander reactions: A case study in the Taiwanese workplace. Asia Pacific Journal of Human Resources.

[B75-behavsci-16-01191] Xiang Z., Zhu Y., Zhang Q., Chen E., Wu X. (2026). The influence of social relationships on third-party punishment: The roles of relationship type congruence and threat perception. Behavioral Sciences.

[B76-behavsci-16-01191] Xu E., Huang X., Jia R., Xu J., Liu W., Graham L., Snape E. (2020). The “evil pleasure”: Abusive supervision and third-party observers’ malicious reactions toward victims. Organization Science.

[B77-behavsci-16-01191] Yang J., Mossholder K. W., Peng T. K. (2007). Procedural justice climate and group power distance: An examination of cross-level interaction effects. Journal of Applied Psychology.

[B78-behavsci-16-01191] Yu Y., Li Y., Xu S. T., Li G. (2022). It’s not just the victim: Bystanders’ emotional and behavioural reactions towards abusive supervision. Tourism Management.

[B79-behavsci-16-01191] Zhang Y., Liao Z. (2015). Consequences of abusive supervision: A meta-analytic review. Asia Pacific Journal of Management.

[B80-behavsci-16-01191] Zhang Y., Liu X., Chen W. (2020). Fight and flight: A contingency model of third parties’ approach-avoidance reactions to peer abusive supervision. Journal of Business and Psychology.

[B81-behavsci-16-01191] Zhang Y., Winterich K. P., Mittal V. (2010). Power distance belief and impulsive buying. Journal of Marketing Research.

